# The Determinants of Out-of-Pocket Expenditure in IBD Italian Patients. Results from the AMICI Survey

**DOI:** 10.3390/ijerph17218156

**Published:** 2020-11-04

**Authors:** Matteo Ruggeri, Carlo Drago, Chiara Cadeddu, Alessandro Armuzzi, Salvo Leone, Marco Marchetti

**Affiliations:** 1 National Center for HTA, Istituto Superiore di Sanità, 00161 Rome, Italy; marco.marchetti@iss.it; 2 School of Medicine, St. Camillus International University of Health Sciences, 00131 Rome, Italy; 3 Faculty of Economics, Università Niccolò Cusano, 00166 Rome, Italy; c.drago@unicusano.it; 4 School of Medicine, Università Cattolica del Sacro Cuore, 00168 Rome, Italy; chiara.cadeddu@unicatt.it (C.C.); alessandro.armuzzi@unicatt.it (A.A.); 5 Fondazione Policlinico Universitario “A. Gemelli” IRCCS, 00168 Rome, Italy; 6 Amici Italia Onlus, 20125 Milan, Italy; salvo.leone@amiciitalia.net

**Keywords:** out of pocket expenditure, equity and quality of care, IBD

## Abstract

Decision makers are used to consider Out-of-Pocket Expenditure (OOPE) within a health technology assessment framework in order to account for an indicator relying on the level of fairness and on the quality of care of a health system. In this paper, we provide estimates on the determinants of OOPE in Italy by using data coming from an observational cross-sectional study that enrolled a sample of 2526 patients suffering from inflammatory bowel diseases. We explore the association between OOPE and: (1) geographical location; (2) income effects; (3) performances in delivering healthcare. A regression model was used. Individuals’ age were in the range of 18–88 (mean 44 ± 14.55). Forty-six percent were females, 54% were married and 19% held a bachelor degree. Ninety-six percent of respondents declared an OOPE >0 whose mean value was €960 ± €950. Individuals belonging to low-income and low-performance regions were more likely to declare an OOPE >0 (99%). Regression findings suggest that increases in OOPE could be considered as a response from patients aiming to compensate for lacks and inefficiencies in the public healthcare offers. Policymakers should consider increases in OOPE in patients with Inflammatory Bowel Diseases (IBDs) as an indicator of poor quality of care and poor fairness.

## 1. Introduction

Out-of-pocket expenditures (OOPE) refer to that portion of services, medicines, examinations, and therapies that are not covered by public health systems. A recent study conducted in China [1] showed how private medical insurance was a major protective factor for middle aged and elderly people, in order to compensate for the lack of a national public health scheme. Decision makers are used to consider OOPE within a health technology assessment framework in order to account for an indicator relying on the level of fairness and on the quality of care of a health system. Traditionally, issues related to OOPE have been analyzed with a particular focus on low- and middle-income countries [2], as fees for public and private healthcare impose relevant economic constraints on patients [3,4,5]. For this reason, OOPE has been defined as “the most unequal and inefficient way to fund health care” [5,6].

However, due to public healthcare budget constraints, more complex needs of the population, ageing and the growing expenditure for medical technologies, OOPE has grown with a yearly rate ranging between 3 and 5% in the vast majority of the developed countries in the last decade [1].

Getzen (2000) [7] argued that healthcare is neither “a necessity” nor “a luxury”; it is “both”, since the elasticity of income changes depending on the level of analysis considered. More concretely, the income elasticity of individual health expenditures under insurance is typically near zero or negative, while the elasticity of national health expenditures with respect to national income is typically greater than 1.0.

In light of this, one should ask why OOPE increases in developed countries. One possible explanation could be related to an income elasticity above 1.0 [8], with OOPE thus being a luxury good. The richest individuals and households could see OOPE as necessity for satisfying high-complexity needs not necessarily covered by a public insurer (i.e., dental care, nutritional support etc.). Otherwise, individuals or households could see OOPE as the solution to mitigate organizational failures or inefficiencies or other negative factors (i.e., long waiting lists, low quality of care etc.) influencing the capability of public offers to provide high-level quality of care [9]. In light of this, individuals and households would be willing to pay for OOPE regardless of their income level. For this reason, it is known that a major reliance on OOPE is likely to have a regressive impact on income distribution [5].

Moreover, in high-income countries, socio-economic inequalities and disparities are growing, and in some countries, important socio-economic gradients are observed between different local or regional jurisdictions. In countries like Italy, the National Health Service is organized following a federal structure, so that each regional authority manages its own healthcare budget. Healthcare service, visits, hospital admissions and treatments are provided under a universalistic coverage according to their inclusion on a list that each jurisdiction is required to provide on a mandatory basis (called Livelli Essenziali di Assistenza—LEA). Some argue [3,4,5] that the federal structure of the Italian healthcare delivery could have contributed to enlarging the traditional gap between northern and southern regions. Some data are likely to favor this argument; for example: (1) substantial differences in the regional healthcare performances indicators; (2) different levels of integration of care and implementation of care pathways; and (3) the way different regions involve new health professional roles [10].

Scientific evidence shows that the level of OOPE is strongly associated with socio-economic indicators, such as: (1) the level of development of the reference country; (2) the average population income; (3) institutional and organizational factors; (4) the level of development of a society. Moreover, parameters affecting OOPE might be disease-specific (for instance, acute or chronic), or related to the degree of severity or the level of disability [11].

Despite these evidences, literature investigating the determinants of OOPE remains substantially scant, particularly regarding high-income countries. More specifically, so far no studies have investigated the determinants of OOPE according to different local or regional jurisdictions within the same country. One of the possible explanations given by the literature [1] concerns the scarce empirical basis offered by official statistical institutions to study the determinants of OOPE at the individual level. Furthermore, disaggregated data reporting different categories of OOPEs (i.e., medicines, exams, visits etc.) is extremely scarce.

Inflammatory bowel diseases (IBDs—Crohn’s disease and ulcerative colitis) are multifactorial etiology pathologies characterized by a common immuno-inflammatory pathogenesis and by intestinal and extra-intestinal manifestations [12].

The incidence of IBD is in continuous increase. In Italy, it has been calculated that there are between 150,000 and 200,000 people affected by IBD, with a prevalence of 100 patients out of 100,000 inhabitants for Crohn’s disease and 121 patients out of 100,000 inhabitants for ulcerative colitis. IBDs can be diagnosed at any age, more frequently in patients aged between 15 and 30 years and in those aged between 50 and 70 years, and are more common in northern European countries (ulcerative colitis, 505 per 100,000 people, Crohn’s disease, 322 every 100,000 people). Recently, there has been a noticeable increase in the incidence of these diseases in Mediterranean Europe, probably due to changes in eating habits. In recent years, there has been an evolution of the therapeutic approach to IBD due to the advent of biological drugs, and mainly due to the use of new therapeutic strategies and increasingly widespread personalized treatments. Nowadays, the target of treatment is no longer the remission of symptoms, but the remission of the disease. IBDs present a high impact both on the health system, in terms of hospitalization and pharmacological treatments, and on the society, in terms of absenteeism from work and loss of productivity [12].

In this paper, we will provide estimates on the determinants of OOPE within the Italian regions by using data coming from the “AMICI “ project, an observational cross-sectional study that enrolled a sample of 2526 patients suffering from inflammatory bowel diseases (IBDs—Crohn’s disease and ulcerative colitis) from each Italian region. IBDs can be considered a good field of application to investigate the determinants of OOPE, since they are chronic diseases with an important epidemiological burden and an impact on quality of life and productivity. Furthermore, they are pathologies involving both women and men regardless of the level of education, socio-economic status and job. Finally, they are pathologies in which technological innovation is complex and has experienced important developments in recent years.

## 2. Materials and Methods

After performing a literature search on the MEDLINE engine, we found that as of now, no studies investigating the OOPE determinants of IBD in Italy were published. Thus, the Italian patients association (AMICI ONLUS) agreed to support a cross-sectional observational open-label study, which aimed at collecting information on the consumption of resources, losses of productivity and private expenditure associated with patients with IBDs. The survey was carried out through the publication of a questionnaire to be completed by patients anonymously on the website of the Italian IBDs patients association. Patients included in the survey were required to be registered on the AMICI website for at least one year prior to the starting of the survey. Moreover, in the first part of the questionnaire, a set of questions dealing with specific information regarding the disease was asked, in order to avoid including fake patients. Patients were asked via email to participate in the survey. The questionnaires were also administered to medical departments distributed across all of the Italian regions. The questionnaire included 75 questions divided in six sections:

- Personal data;
- General health status;
- IBDs-related questions;
- Assistance received for IBDs;
- Disease and costs;
- Health organization and patient satisfaction.

The survey was administered between January and December 2017.

In total, 2526 respondents (approximately 1.25% of cases in Italy) participating the survey met the following inclusion criteria: (1) aged more than 18 years; (2) having been diagnosed with one of the IBDs; (3) having received assistance for one of the three diseases under examination: Crohn’s disease and ulcerative or indeterminate colitis. Mean age was 44.

Table 1 shows the main characteristics of the sample.

### 2.1. Hypotheses

From now on, we will refer to W_OOPE_, which occurs if OOPE >0. We aim to explore whether an association exist between W_OOPE_ and: (1) geographical location (i.e., Northern, Central or Southern Italy); (2) region-specific income effects (i.e., regional per capita GDP); (3) region-specific performances in delivering healthcare (i.e., quality of care, efficiency, waiting lists etc.). In order to test our hypotheses we identified a set of covariates from the AMICI dataset, which are coherent with the indications extrapolated from the literature (i.e., proxies of disease severity, demographic variables, education etc.). Next, we clustered Italian regions according to three main criteria: (1) geographic (North, Center or South); (2) income-based (with Italian regions divided into three clusters depending on the per capita GDP) and (3) performance-based.

Concerning the income-based criterion, regions were classified according to the regional per capita GDP as resulting from Italian Statistic Office data [13]. In particular, clusters (1) “high”, (2) “medium” and (3) “low” include regions with per capita GDP in the ranges of the (1) 100th–67th; (2) 66th–34th; (3) 33rd–1st percentiles of the national per capita GDP distribution. Concerning the performance-based criterion, Italian regions were divided into three clusters depending on the IPS—“Indice di Performance Sanitaria” (Health Performance Index) developed by the demoskopika survey in 2017 [14]. The IPS is an index summarizing information about the performance of the Italian health organizations. It is calculated including information on healthcare satisfaction, intra- and extra-region patients’ mobility, waiting lists, costs of policies, expenditure for legal issues, health expenditure and number of families under the poverty threshold, and allows ranking Italian regions into three clusters (high, medium and low organizational performance).

According to geographical criteria, Italian regions were grouped as follows:

- North: Lombardy, Pedemont, Liguria, Veneto, Val d’Aosta, Friuli Venezia Giulia, Trentino Alto Adige, Emilia Romagna;
- Center: Toscana, Umbria, Marche, Lazio;
- South: Campania, Abruzzo, Molise, Basilicata, Calabria, Sicily, Sardinia.

According to the income-based criteria, Italian regions were grouped as follows:

- High: Val d’Aosta, Trentino-Alto-Adige, Lumbardy, Emilia-Romagna, Latium, Veneto, Tuscany;
- Medium: Liguria, Piemonte, Friuli-Venezia Giulia, Marche, Umbria;
- Low: Abruzzo, Molise, Basilicata, Puglia, Campania, Calabria, Sicily, Sardinia;

According to the performance-based criteria, Italian regions were grouped as follows:

- High: Trentino-Alto-Adige, Emilia-Romagna, Lumbardy, Val d’Aosta, Marche, Veneto;
- Medium: Tuscany, Umbria, Latium, Liguria, Friuli-Venezia Giulia;
- Low: Basilicata, Puglia, Molise, Calabria, Campania, Sicily, Sardinia.

### 2.2. Empirical Strategy

The most commonly used approach in the literature [15] to estimate parameters affecting W_OOPE_ is the two-stage Heckman decomposition, which allows taking into account the typically censored distribution of health expenditure.

More in detail, the use of such an approach allows us to select our sample depending on the W_OOPE_ being higher than zero. In fact, the Italian National Health Service is public-funded, and patients receive care they need on a universalistic basis. As the IBDs are included in the LEAs, our expectation was to find zero responses from individuals for which W_OOPE_ is not observed. We followed the canonical specification of the Heckman decomposition. In the first stage, we estimated the selection equation through a probit model:

(1) $$Prob\;\left(D_{H}|DEM;EDU\right)=\;\Phi \left(Z_{\gamma }\right)$$

where *D_OOPE_* represents a dummy variable indicating whether *OOPE* is observed *(D_OOPE_* = 1) or not (*D_OOPE_* = 0). We consider *(DEM; EDU)* as a set of covariates explaining demographic characteristics and level of education, *Z* a vector of unknown parameters and φ the cumulative distribution function of a normal random variable. We present the selection equation in an extended form as:

*D_OOPE_ = α + β_1_AGE + β_2_AGE+ β_3_AGE^2^ + β_3_ BACHELOR + β_4_ FEMALE+ ε* (2)

(3) $$D=+AGE+b2\;AGE^{2}+b3BACHELOR+b4FEMALE+e$$

In the second stage, we correct for self-selection by incorporating an additional explanatory variable, a transformation of the probability of *D* = 1 estimated in the first stage. The *W_OOPE_* equation can be specified as:

*E[W_OOPE_| (DEM;EDU;DIS;REG), D_OOPE_ = 1] = X + E [(DEM; EDU; DIS), D_OOPE_ = 1]* (4)

where (*DEM; ED; DIS; REG*) is a set of controls explaining individual demographic, educational and disease-specific covariates explaining severity and region-specific characteristics. We assume that the error terms in both equations are jointly normal, and thus we can rewrite (5) as:

*E[W_OOPE_| (DEM;EDU;DIS;REG), D_OOPE_ = 1] = (DEM; EDU; DIS; REG) + σ_υ_λ(Z_γ_)* (5)

We can write the *W_OOPE_* equation in expanded form as:

*W_OOPE_ = γ_1_ + γ_2_ AGE + γ_3_ AGE^2^ + γ_4_ FEMALE + γ_5_ BACHELOR + γ_6_ SELFCARE + γ_7_ RELAPSE+ γ_8_ SURGERY + γ_9_ NUTSUP + γ_10_ CORTIST + γ_11_ OTHER + γ_12_ REG(CRITERION)_CLUSTER1_ + +γ_13_ REG(CRITERION)_CLUSTER2_ + σ_u_λ(Z_γ)_* (6)

where:

*AGE*, *AGE*^2^ and *FEMALE* explain demographic effects;

*BACHELOR* explains education levels;

*SELFCARE*, *RELAPSE*, *SURGERY*, *NUTSUP*, *CORTIST* and *OTHER* are disease-specific variables explaining severity.

Table 2 describes all of the variables used to estimate both the selection and the monetary OOPE equations.

The W_OOPE_ Equation (6) was estimated according to different specifications of *REG (CRITERION)* variables, identifying the different criteria for regional clustering (geographic, per capita income-based and organizational performance-based). The subscripts CLUSTER 1 and 2 identify different clusters of regions according to the followed clustering criterion, in detail:

- Northern and southern regions for the geographical criterion;
- Regions with high and medium per capita income for the per capita income criterion;
- Regions with high and medium organizational performance for the organizational performance criterion.

#### Bootstrap Analysis

A multi-way bootstrap analysis was conducted in order to investigate the variability and the generalizability of the results. Bootstrapping is a statistical approach allowing to simulate alternative resamples starting from a single dataset. This process allows to investigate the robustness of original estimates, namely the ones resulting from our regression [16]. One-thousand iterations were performed, where all the covariates of the model were subject to a resampling procedure assuming a skewed distribution, which is coherent with the Heckman assumptions.

One-way bootstraps were performed in order to observe the elasticity of the W*_OOPE_* estimation according to the variation of age and number of relapses. Finally, scenario bootstrap simulations were performed in order to observe potential differences according to regional clusters.

In all bootstrap simulations, the 10th, 25th, 50th, 75th and 90th percentiles are presented in tabular format.

## 3. Results

### 3.1. Sample Characteristics and OOPE Estimates

Individuals’ ages were in the range of 18–88 (mean 44 ± 14.55). Forty-six percent were females, 54% were married and 19% held a bachelor degree. Ninety-six percent of respondents declared an OOPE >0 (see Table 3) whose mean value was €960 ± €950. According to the clustering criteria described above, individuals belonging to low-income and low-performance regions were found to be more likely to declare an OOPE >0 (99%). Moreover, these individuals were also associated the highest OOPE both in terms of mean (€1064 ± 1411 and €1071 ± 1259, respectively) and maximum values (€3000 in both cases). Conversely, individuals aged >64 were associated with the lowest percentage of declaring an OOPE >0 (89%). Individuals aged 50–64 were associated with the lowest OOPE values both in terms of mean (€563 ± €665) and maximum values (€1160).

On average, females were observed to have a higher OOPE compared to males across all percentiles. This was found to be statistically significant. Concerning education, no relevant differences were observed across percentiles related to patients holding a bachelor degree compared to others, nor were they found to be statistically significant. Concerning age, OOPE values were observed to decrease while age increases. The only exception is represented by the 90th percentile where a non-constant trend was observed, and the value of the 90th percentile was the highest for the age group >64.

Patients belonging to northern regions exhibited a lower OOPE compared to central and southern inhabitants. This difference was observed across all percentiles and was statistically significant. The same results were observed when considering the income- and performance-based regional clustering, as regions belonging to the highest subgroups showed the lowest OOPE values.

### 3.2. Regression Analysis

Table 3 shows the results of the regression analysis performed using Heckman’s decomposition. Three models are presented, each incorporating one different criterion for clustering.

Table 3 shows the results of the selection equations. Significant coefficients were found concerning age (*p* < 0.05 in all three models) and age squared (*p* < 0.01 in all three models). In particular, whilst the relation with age was positive, the age-squared coefficient was negative. Concerning education, a positive and significant coefficient was found for patients holding a bachelor degree exhibiting a higher W_OOPE_ in all three models (*p* < 0.01). Gender was not found to be significant.

Table 3 shows the results of the W_OOPE_ estimation across the three estimated models. Non-significant inverse relations with age and gender were found, with patients more likely to decrease W_OOPE_ as they get older or if they are females. A positive relation was observed for the level of education (*p* < 0.05 in the geographical clustering criterion and *p* < 0.1 in the others) with patients holding a bachelor degree exhibiting higher levels of W_OOPE_. Again, higher W_OOPE_ is likely to be strongly related to the decrease of functioning in self-care (*p* < 0.01 in all three models) and as patients experience relapses (*p* < 0.01 in all three models), which can be assumed as a proxy for severity. Again, W_OOPE_ was higher in patients that experienced surgery (*p* < 0.01 in all three models), that were enrolled in nutritional support programs (*p* < 0.01 in all three models), that were administered corticosteroids (*p* < 0.01 in all three models) and with treatments concerning other diseases (*p* < 0.01 in all three models).

Concerning the geographical clustering criterion, our results show that patients receiving care in northern (*p* < 0.01) and central (*p* < 0.05) regions were likely to exhibit a lower W_OOPE_ compared to patients being treated in the southern regions.

When an income-based clustering criterion was used, patients were found to be likely to exhibit a lower W_OOPE_ when receiving care in regions with high (*p* < 0.01) and medium (*p* < 0.01) per capita GDP.

Finally, W_OOPE_ was likely to decrease in regions ranked higher in the performance-based criterion (*p* < 0.01). A non-significant relationship was found concerning regions included in the medium level of the performance cluster.

### 3.3. Bootstrap Analysis

Concerning the multi-way analysis, the OOPE estimation ranged between €601 and €792, with the 10th–90th percentile values ranging between €654 and €730.

The results of the one way bootstrap simulations are reported in Table 4. For each variable included in the model, the 10th to 90th percentiles of the OOPE are fitted. The highest variability can be found with regard to the southern regions and the regions that had a lower performance index and income, thus confirming the results of the Hackman regression.

Moreover, participants aged >64 were observed to be associated with the highest variability compared to the other age classes.

Given this, when observing the 50th percentile, participants aged <25 and 25–39 were found to be associated with the highest values. In general, females showed higher OOPE values than males. No significant differences were observed depending on the education level.

## 4. Discussion

In this paper, we studied the determinants of OOPE in Italian patients suffering from IBDs.

We used data from a cross-sectional, observational survey involving 2526 patients from all of the Italian regions. Due to their high heterogeneity, Italian regions were clustered according to three criteria: geographic, income- and performance-based. The Heckman decomposition showed how, controlling for age, education and severity, OOPE is likely to increase in southern regions and where per capita income and the performance of the health care services are lower.

Our research is affected by some limits. Firstly, our model does not take into account any spatial autocorrelation. Nevertheless, we ran some preliminary analyses and did not find any potential spatial effects that would have affected our results. In addition, we included in the covariates the distance in kilometers that patients declared having to cover in order to reach their healthcare services and we did not find significant associations.

Secondly, our results are based on a cross-sectional survey, which does not allow investigating for causal effects. Moreover, the OOPE amounts were self-reported, which, in principle, would increase the probability of having reverse causality effects. However, given the basic assumptions of our model, we believe that these effects would be very limited. Moreover, we accurately avoided including in our covariates subjective information, such as quality of life, patients’ engagement and patients’ satisfaction coefficients.

Thirdly, our model does not account for any potential association between OOPE and the inclusion of new health professional roles (i.e., nursing staff, pharmacists, nutritionists) in the care pathway, which is to be considered a relevant issue in the chronic care field and in general in the reorganization of the healthcare services. Currently, the introduction in the Italian regions of new health professional roles in the care pathways is very heterogeneous [16,17] but the managing of care is mostly physician-centered. For this reason, it would be very difficult to identify a criterion that can be used to cluster regions according to the level of involvement of new health professional roles. Again, our data included specific information. In our preliminary analyses, we tried to include in the model a covariate explaining whether patients had seen a nurse or not during their pathway of care, but we did not find significant effects, with almost all patients declaring to have seen a nurse at least one time. The reason can be explained by considering that when undergoing ambulatory care patients or surgery patients see nurses, which does not mean that new professional roles, such as, for instance, disease managers, are involved in their care pathway. Nevertheless, the potential association of OOPE with the involvement of new health professional roles, as well as the association with patients’ satisfaction and levels of engagement, remain important issues that deserve to be investigated in future works.

In this paper we studied the association between OOPE and a set of covariates. However, an important issue for discussion regards the generalizability of both the approach and the results of the model.

In this regard, it is important to reiterate that the survey from which we extrapolated the data refers to a particular class of diseases, the IBDs. It is understood that any attempt to generalize our approach and results, by extending them as they are to other pathologies, would be unrealistic.

Another limit of our study concerns the fact that data were extrapolated from a survey among IBD patients, and therefore pour model was entirely structured based on self-reported data, which could generate issues associated with endogeneity. There is currently a part of the literature that uses non-subjective sources of data, for example internet search queries (see for example [18]). However, this methodology seems more appropriate to estimate volumes of resource consumption. Instead, the goal of the present paper was to estimate gradients in the amounts of OOPE.

With particular regard to the methodology used, we would like to support the idea that, with appropriate readjustments, it can be replicated for other diseases, as Heckman’s model is used very extensively where the research question has to do with estimating censored data, such as costs.

However, alternative methodologies could be used in order to account for issues regarding heterogeneity or endogeneity that are typically associated with traditional regression techniques.

Lin et al. (2020), for example, used machine learning techniques in order to forecast demand for ambulance services in Singapore [19]. In future studies, the authors will consider applying similar techniques in order to predict OOPE expenditure and to better investigate possible gradients that are associated with spatial location, income etc. More in detail, machine learning techniques could be used in order to test for the possible reorganization of the healthcare deliveries in geographical locations where OOPE levels are higher than the national average, and find the most efficient interventions aimed at improving quality of care and minimizing waste of resources.

A second consideration concerns the type of controls that were used in the model. Obviously, beyond the demographic variables, we chose IBD-specific controls to include in our model information related to severity, the presence of comorbidity, and having undergone surgical interventions that are typically foreseen in the natural history of the IBDs where the pathology progresses. In addition, the choice of type of drugs (corticosteroids) was due to the need to model the OOPE of a specific pathology. In these cases, in order to make our approach also applicable to other diseases, it would be necessary to choose other variables. For example, in the case of cardiovascular diseases, the variable relating to surgery could be replaced with a variable that explains the number of events or the type of intervention (bypass or angioplasty) received. In the case of other inflammatory pathologies, such as psoriasis or rheumatoid arthritis, the choice of the variables related to corticosteroid drugs could be maintained as an alternative to the use of biological drugs.

A separate consideration is the inclusion of a variable concerning nutritional support. In the case of IBDs, this was important since these interventions are typically prescribed in a manner coherent with personalized treatment plans and are increasingly emphasized by clinicians. Furthermore, since food for nutritional support is not reimbursed by the Italian public health system, we considered it as a potential significant predictor for the OOPE estimate. In the case of other diseases, researchers should think about what could be the type of support or care that is not reimbursed but is a crucial element in the management of the disease.

An issue per se regards the case in which the researcher finds him- or herself having to work on a dataset containing information about patients with different pathologies. At that point, if on the one hand the accuracy of the estimates and their generalizability would increase, on the other hand it would be crucial to choose a criterion to select disease-specific variables for each pathology that are comparable and consistent with the research question. Excessive use of dummy variables could burden the model and compromise the significance of the results. The interval data approach could also be useful in this case. Nevertheless, we think that our basic assumptions and clustering criteria can be maintained also when investigating other disease areas.

Clearly, it must be said that when dealing with multi-country data, researchers should not disregard the particular characteristics of the countries’ respective health systems. The type of funding as well as the organization or other types of institutional variables could be critical in choosing the variables to be used in the model. For example, if in some countries involved in the analysis a form of co-payment is associated with income or if exemptions for particular categories of workers are provided, these elements should be taken into account.

Regardless of this, however, if the goal is to estimate the OOPE concerning patients from different countries, one should also choose classification criteria that are different from the ones we have chosen.

## 5. Conclusions and Policy Implications

Our findings suggest that in Italy, increases in OOPE could be considered as a response from patients aiming to compensate for lacks and inefficiencies in the public healthcare deliveries. Thus, policymakers should consider increases in OOPE as an indicator of poor quality of care and difficulty accessing care.

Therefore, policies aimed at facilitating access and improving public healthcare should be considered as an instrument to reduce inequalities.

## Figures and Tables

**Table 1:** Sample characteristics.

Characteristics	(%)	Average	MIN	MAX	SD
Aged > 44	52%	44	11	88	14.55
Female	46.61%	-	-	-	-
Married	53.94%	-	-	-	-
High school diploma	49.03%	-	-	-	-
Bachelors degree	18.52%	-	-	-	-
Worker	58.54%	-	-	-	-
Diagnosed with Crohn’s disease (CD)	52%	-	-	-	-
Treated with Corticosteroids (Crohn’s disease)	55%	-	-	-	-
Treated with corticosteroids (ulcerative colitis)	51%	-	-	-	-
Treated with biologics (Crohn’s disease)	29%	-	-	-	-
Treated with biologics (ulcerative colitis)	18%	-	-	-	-
Undergone surgery (Crohn’s disease)	12%	-	-	-	-
Undergone surgery (ulcerative colitis)	7%	-	-	-	-
Experiencing remission (Crohn’s disease)	68%	-	-	-	-
Experiencing remission (ulcerative colitis)	71%	-	-	-	-
Lost days of work	78.68%	27	0	365	50
Lost days of work (Caregiver)	61.52%	11	1	365	23

**Table 2:** Variables included in the model. OOPE: Out-of-Pocket Expendture; IBD: Inflammatory Bowel Disease

Variable	Independent	Selection Equation	Type	Description	Values
W_OOPE_	NO	NO	Continuous	Monetary values (€) of the OOPE	€0–€23.610
D_OOPE_	NO	YES	Dummy	DOOPE = 1 if OOPE is observed	0; 1
AGE	YES	YES	Continuous	Age of individuals	18–76
BACHELOR	YES	YES	Dummy	Level of education of each individual	1 = higher than bachelor; 0 = otherwise
FEMALE	YES	YES	Dummy	FEMALE = 1 if individual is female	0; 1
SELFCARE	YES	NO	Dummy	SELFCARE = 1 if individuals face problems in taking care of themselves	0; 1
RELAPSE	YES	NO	Dummy	RELAPSE = 1 if individuals experienced at least one relapse in the last year	0; 1
SURGERY	YES	NO	Dummy	SURGERY = 1 if individuals underwent IBD-related surgery	0; 1
NUTSUP	YES	NO	Dummy	NUTSUP = 1 if individuals have been prescribed some nutritional support (not reimbursed by the Italian National Health Service)	0; 1
CORTIST	YES	NO	Dummy	CORTIS = 1 if individuals have been administered with corticoseroids, which involve more side effects and may involve the use of additional treatments	0; 1
OTHER	YES	NO	Dummy	OTHER = 1 if individuals are currently undergoing other treatment for other diseases	0; 1
REG(CRITERION)cluster1,2	YES	NO	Dummy	REG (CRITERION) cluster1 = 1 if individuals belong to cluster 1 or 2 of the selected criterion	0; 1
CRITERION	n.a.	n.a.	n.a.	Criteria adopted to classify regions in different specifications	Geographical, per capita income, organizational performance
cluster1 and 2	n.a.	n.a.	n.a.	Clusters identified to include regions according to selected criterion	Geographic criterion: Cluster 1 = North Cluster 2 = Center Income and performance-based criterion Cluster 1 = High Cluster 2 = Medium

**Table 3:** Parametric analysis.

Selection Equation			
Criteria			
Variables	Geographic	Income-Based	Performance-Based
AGE	0.025 **	0.21 **	0.22 **
AGE-SQUARED	−0.00047 ***	−0.00042 ***	−0.00042 ***
FEMALE	0.56	0.27	0.51
BAHELOR	0.27 ***	0.25 ***	0.25 ***
Constant	0.45 **	0.63 ***	0.66 ***
**W_OOPE_ Equation**			
AGE	−0.021	−0.0019	−0.002
AGE-SQUARED	−0.0026	−0.0067	−0.013
FEMALE	0.025	0.012	0.011
BAHELOR	0.078 **	0.064 *	0.060 *
SELF CARE	−0.30 ***	−0.31 ***	−0.31 ***
RELAPSE	0.067 ***	0.079 ***	0.08 ***
SURGERY	0.31 ***	0.27 ***	0.27 ***
NUTSUP	0.47 ***	0.45 ***	0.45 ***
CORTIST	0.17 ***	0.16 ***	0.17 ***
OTHER	0.20 ***	0.17 ***	0.18 ***
REG(GEOGRAPHICAL)_NORTH	−0.23 ***		
REG(GEOGRAPHICAL)_CENTER	−0.15 **		
REG(INCOME BASED)_HIGH		−0.19 ***	
REG(INCOME BASED)_MEDIUM		−0.13 ***	
REG(PERFORMANCE BASED)_HIGH			−0.15 ***
REG(PERFORMANCE BASED)_MEDIUM			−0.03
Constant	5.19 ***	5.34 ***	5.34 ***
ATHAN-RHO	0.08	0.042	0.06
LN SIGMA	−0.17 ***	−0.17 ***	−0.16 ***
N	1.356	1.551	1.551

Legend: * *p*(t) < 0.10; ** *p*(t) < 0.05; *** *p*(t) < 0.01.

**Table 4:** Bootstrap results.

Percentile	Males	Females	Bachelor	No Bachelor	Age <25	Age 25–39	Age 40–49	Age 50–64	Age >64	North	Center	South	Income High	Income Medium	Income Low	Hpi High	Hpi Medium	Hpi Low
10th	10	15	5.55	17.4	15.2	12.8	6.2	17.8	7.2	7	5.04	5.6	2.65	10	2.95	2.2	7.4	2.95
25th	175	200	200	200	200	200	200	200	100	167.5	200	200	159	150	250	156	220	250
50th	350	400	400	400	450	450	353.5	350	300	350	400	400	350	305	500	325	450	500
75th	750	900	900	800	800	900	900	700	750	800	800	800	800	750	1000	800	800	1000
90th	1500	1600	1500	1500	1500	1500	1650	1160	2000	1500	1680	2000	1500	1500	3000	1500	1500	3000
